# Isolation and functional analysis of phage‐displayed antibody fragments targeting the staphylococcal superantigen‐like proteins

**DOI:** 10.1002/mbo3.1371

**Published:** 2023-07-16

**Authors:** Ida Alanko, Rebecca Sandberg, Eeva‐Christine Brockmann, Carla J. C. de Haas, Jos A. G. van Strijp, Urpo Lamminmäki, Outi M. H. Salo‐Ahen

**Affiliations:** ^1^ Faculty of Sciences and Engineering, Pharmaceutical Sciences Laboratory (Pharmacy) & Structural Bioinformatics Laboratory (Biochemistry) Turku Åbo Akademi University Turku Finland; ^2^ Department of Life Technologies University of Turku Turku Finland; ^3^ Department of Medical Microbiology, University Medical Center Utrecht Utrecht University Utrecht The Netherlands

**Keywords:** antivirulence strategy, phage display, scFv, staphylococcal superantigen‐like protein, *Staphylococcus aureus*

## Abstract

*Staphylococcus aureus* produces numerous virulence factors that manipulate the immune system, helping the bacteria avoid phagocytosis. In this study, we are investigating three immune evasion molecules called the staphylococcal superantigen‐like proteins 1, 5, and 10 (SSL1, SSL5, and SSL10). All three SSLs inhibit vital host immune processes and contribute to *S*. *aureus* immune evasion. This study aimed to identify single‐chain variable fragment (scFvs) antibodies from synthetic antibody phage libraries, which can recognize either of the three SSLs and could block the interaction between the SSLs and their respective human targets. The antibodies were isolated after three rounds of panning against SSL1, SSL5, and SSL10, and their ability to bind to the SSLs was studied using a time‐resolved fluorescence‐based immunoassay. We successfully obtained altogether 44 unique clones displaying binding activity to either SSL1, SSL5, or SSL10. The capability of the SSL‐recognizing scFvs to inhibit the SSLs' function was tested in an MMP9 enzymatic activity assay, a P‐selectin glycoprotein ligand 1 competitive binding assay, and an IgG1‐mediated phagocytosis assay. We could show that one scFv was able to inhibit SSL1 and maintain MMP9 activity in a concentration‐dependent manner. Finally, the structure of this inhibiting scFv was modeled and used to create putative scFv‐SSL1‐complex models by protein–protein docking. The complex models were subjected to a 100‐ns molecular dynamics simulation to assess the possible binding mode of the antibody.

## Graphical abstract

In this study, we report the identification of phage‐displayed scFv‐antibodies able to recognize the staphylococcal superantigen‐like proteins SS1, SSL5, and SSL10. All three SSLs bind to and inhibit host immune components and contribute to *Staphylococcus aureus* immune evasion. We were able to show in vitro that one scFv was able to inhibit SSL1 and maintain matrix metallopeptidase (MMP9) activity in a concentration‐dependent manner.

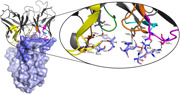

## 1 INTRODUCTION

*Staphylococcus aureus* is a Gram‐positive bacterium that colonizes one‐third of the population (Lowy, 1998). Carriers are often asymptomatic; however, *S*. *aureus* is an opportunistic pathogen and colonized individuals are at an increased risk of acquiring staphylococcal bacteremia (Wenzel & Perl, 1995; Wertheim et al., 2004). The significance of this is further highlighted during the SARS‐CoV‐2 pandemic as bacterial copathogens are common among the infected, and it has been reported that as much as half of the nonsurvivors experienced a secondary infection (Zhou et al., 2020). In addition, hospital‐admitted patients are further at a higher risk of being exposed to resistant bacterial strains, such as methicillin‐resistant *Staphylococcus aureus* (MRSA) (Lakhundi & Zhang, 2018; Tajeddin et al., 2016).

In part, *S*. *aureus'* success as a pathogen is explained by its arsenal of so‐called immune evasion molecules, virulence factors that specifically focus on evading the host immune system (de Jong et al., 2019). To date, around 40 molecules that interact with components of both the adaptive and innate immune systems have been identified in *S*. *aureus*, making it a record for any bacterium. It has been argued that immune evasion is one of the reasons why vaccination attempts against *S*. *aureus* have failed (de Jong et al., 2019). This is because the secreted evasion molecules target the effector system that is required for the vaccine‐induced antibodies to provide adequate protection. Therefore, inhibiting the evasion molecules with antivirulence agents could provide a solution to the efficacy issues of vaccines in the future. Such antivirulence strategies may also provide a useful alternative to antibiotics (Hotinger et al., 2021) or at least, effective adjunctive therapy to support the antibiotic treatment of patients with severe infections caused by resistant bacterial strains (Dehbanipour & Ghalavand, 2022). For example, there is *S*. *aureus* virulence factor neutralizing antibodies currently under clinical investigation. The most notable ones are the two monoclonal antibodies, MEDI4893 (suvratoxumab) and AR‐301 (tosatoxumab), against the *S*. *aureus* pore‐forming toxin α‐hemolysin (Hla) (François et al., 2018; Yu et al., 2017) both of which have reached Phases 2 and 3 clinical trials, respectively. In addition, various preclinical investigations against *S. aureus* virulence factors are actively ongoing (Theuretzbacher et al., 2020).

Phage display is a well‐established method where, for example, antibodies (or antibody fragments) are expressed in the phage coat protein (Bradbury et al., 2011). A phage library with billions of varying antibodies can be repeatedly subjected to an immobilized target antigen to steer the selection toward more target‐specific antibodies (Frenzel et al., 2016). Antibody phage display enables the identification of human antibodies more rapidly and efficiently, without the need to rely on immunity, and the human antimouse antibody reaction caused by the hybridoma technology is minimized (Zhao et al., 2023). Compared to complete antibodies, smaller antibody fragments can have potentially beneficial properties, such as better penetration to tissues or access to challenging epitopes (Power & Bates, 2019). For example, selective single chain variable fragment (scFv) antibodies against *S*. *aureus* toxins, such as toxic shock syndrome toxin‐1 (TSST‐1) (Rukkawattanakul et al., 2017) and Hla (Foletti et al., 2013; Xu et al., 2022) have been obtained using the phage display technology. The anti‐TSST‐1 scFvs were shown to reduce the toxin effects in cell‐based assays. On the other hand, the anti‐Hla scFvs were first converted to full antibodies, and their anti‐virulence effects were confirmed by cell‐based functional assays.

**Figure 1:**
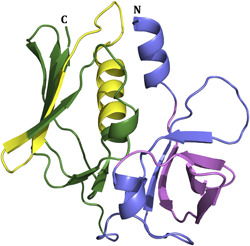
Crystal structure of SSL1 (PDB ID: 4O1N). The N‐terminal oligosaccharide‐binding (OB) domain is colored slate blue, and the C‐terminal β‐grasp domain is green. The SSL1 N‐terminal β1‐3 domain and the C‐terminal α4β9 domain are colored violet and yellow, respectively.

Staphylococcal superantigen‐like proteins (SSLs) are an interesting family of structurally related immune evasion molecules in the *S. aureus* repertoire of virulence factors. SSLs are composed of an N‐terminal oligosaccharide‐binding (OB) domain and the C‐terminal β‐grasp domain (Figure 1) similar to the superantigens (sAgs such as TSST‐1) but SSLs exhibit no superantigenic activity (Williams et al., 2000).

The SSL family includes 14 highly conserved proteins named SSL1‐SSL14, each involved in the innate immune system evasion through different targets (Bardoel et al., 2012; Bestebroer et al., 2007; Chung et al., 2007; Itoh et al., 2010a, 2017; Koymans et al., 2015; Koymans et al., 2016; Laursen et al., 2010; Patel et al., 2017; Ramsland et al., 2007; Tang et al., 2019; Walenkamp et al., 2010; Wines et al., 2011; Yang et al., 2022; Yokoyama et al., 2012; Zhao et al., 2018). SSLs are grouped depending on the presence of a sialylated glycan binding site. This conserved motif is located at the β‐grasp domain in SSL2‐6 and SSL11 and binds to the sialyl Lewis X (sLe^*x*^) tetrasaccharide, an endogenous antigen on the surface of immune cells (Baker et al., 2007; Chung et al., 2007; Hermans et al., 2012; Yang et al., 2022). Targets and the immune evasive functions for most SSLs have been identified. SSL1 and SSL5 are both broad‐range matrix metalloproteinase (MMP) inhibitors, including MMP8 and MMP9 that facilitate neutrophil flow to the site of infection (Koymans et al., 2016). Also, SSL5 and SSL11 bind to P‐selectin glycoprotein ligand‐1 (PSGL‐1) and block its binding to P‐selectin, thus inhibiting neutrophil rolling (Bestebroer et al., 2007; Chung et al., 2007). In addition to its immunoevasive function, dimeric SSL1 has also recently been found to cleave collagens and cytokines in vitro and mediate corneal toxicity (Tang et al., 2019). SSL3 and SSL4 block Toll‐like receptor 2 (TRL2) activation by bacterial lipopeptides, though SSL3 far more strongly than SSL4 (Bardoel et al., 2012; Koymans et al., 2015). SSL7 binds to both IgA and complement factor C5 with a high affinity blocking the cleavage of C5 and IgA binding to FcαR (Laursen et al., 2010; Lorenz et al., 2013; Ramsland et al., 2007). SSL8 inhibits extracellular matrix protein tenascin C (TNC) from interacting with fibronectin, leading to weakened motility of keratinocytes (Itoh et al., 2013). SSL10 inhibits bacterial recognition by blocking the binding of IgG1 to FcγR (Itoh et al., 2017; Patel et al., 2010). Contrary to other SSLs, which inhibit immune components, SSL13 induces neutrophil activation specifically via formyl‐peptide receptor 2 (FPR‐2). However, SSL13 may have additional functions that are yet to be uncovered (Zhao et al., 2018).

The SSL family provides an interesting target for antibacterial drug development since it is relevant for a large spectrum of *S. aureus* lineages, including both human and animal isolates (Fitzgerald et al., 2003; Kuroda et al., 2001; McCarthy & Lindsay, 2013; Smyth et al., 2007). In addition, the expression of the SSLs is upregulated under cell stress: when exposed to human whole blood and during hemin‐mediated membrane damage (Attia et al., 2010; Malachowa et al., 2011). The main objective of this study was to use phage display to obtain scFv antibodies that would be specific to three SSLs: SSL1, SSL5, or SSL10. The choice of the three SSLs as antigens was based on our prior knowledge about their human ligands and the functional tests that we had at hand to study the effect of the identified scFvs on the function of these virulence factors. In addition, we were interested in shedding light on the detailed binding interactions of these SSLs and their human ligands as these have not yet been elucidated. Moreover, we wanted to include a variety of SSLs in our study, including sialylated glycan binding (SSL5) and nonbinding (SSL1 and SSL10) proteins. Of note, SSL1 and SSL5 proteins are more closely related to each other than they are to SSL10 (based on sequence similarity) (Figure A3), which was ideal for our initial purpose of investigating potential cross‐reactivity among SSLs. We aimed to obtain scFvs that would inhibit their SSL targets from binding to their above‐mentioned binding partners (human immune system proteins). Inhibitory antibodies could eventually be used as antivirulence agents against *S. aureus* infections as adjunctive therapies with conventional antibiotics to help restore the capacity of the immune system and further enable a more efficient clearance of the bacteria.

Here we report the identification of 44 antibodies able to recognize either of the three SSLs. In addition, we conducted some well‐defined functional analyses on the SSL‐binding scFvs and found antibodies able to inhibit SSL5 and SSL1 functions.

## 2 MATERIALS AND METHODS

### 2.1 Synthetic antibody library and targets

Two different synthetic scFv phage libraries, scFvM, and scFvP designed to target especially haptens and proteins, respectively, were used for panning (Huovinen et al., 2013). Both libraries have been shown to give low nanomolar affinity binders to protein targets (Huovinen et al., 2013). Three different SSLs were used as targets: SSL1, SSL5, and SSL10. The targets were provided by Prof. Jos A. G. van Strijp's group at UMC Utrecht, the Netherlands. SSLs were biotinylated (EZ‐Link NHS‐PEG4‐Biotin; Thermo Fisher Scientific) and the buffer was exchanged to TBS using NAP^TM^ columns following the manufacturer's instructions (GE Healthcare Life Sciences).

### 2.2 Panning

The scFvM and P libraries were mixed and selected against SSL1, SSL5, and SSL10 by three rounds of panning using paramagnetic beads (M280 streptavidin Dynabeads; Invitrogen). The experiments were carried out at room temperature unless otherwise stated. The panning experiments were conducted separately for each antigen in the same manner, alternating between neutravidin and streptavidin‐coated beads between panning rounds to reduce enrichment towards the bead coating. To further reduce unspecific binding, before each panning round the phages were subjected to a negative selection using streptavidin and biotin without the target proteins.

The actual panning was conducted by immobilizing the biotinylated antigens 8 μg SSL1, 20 μg SSL5 and 2.8 μg SSL10 on 0.5 mg beads. In the 2nd and 3rd panning round 0.05 mg of beads were saturated with 0.8 μg SSL1, 2 μg SSL5, and 0.28 μg SSL10. 5 × 10^12^ cfu of phages (10^11^ cfu in the 2nd and 3rd panning round) diluted in TBT‐ 0.05 (50 mM Tris, 150 mM NaCl, 1% bovine serum albumin [BSA], 0.05% Tween‐20, pH7.5) were blocked with biotin, and incubated for 2 h with the beads. The beads were then washed thrice with TBT‐0.05 and once with tris‐buffered saline (TBS). The bound phages were eluted with 60 μg/mL trypsin (Sigma‐Aldrich) for 30 min. The trypsin was inhibited by adding 50 μg/mL soybean trypsin inhibitor (Sigma‐Aldrich).

The phage eluate was used to infect *Escherichia coli* XL1‐Blue cells. The cells were grown to the logarithmic growth phase and infected with the phages for 30 min at 37°C. The infected culture was plated on LA plates (0.5% glucose, 10 μg/mL tetracycline, and 25 μg/mL chloramphenicol; Sigma‐Aldrich) and incubated at 30°C overnight. In addition, the output for the panning was monitored by spreading serially diluted infected cultures on LA plates (0.5% glucose, 10 μg/mL tetracycline, 25 μg/mL chloramphenicol) and incubated at 37°C overnight. The formed cell colonies were then diluted to an OD_600_ of 0.1 in a fresh SB medium (1% glucose, 10 μg/mL tetracycline, and 25 μg/mL chloramphenicol). After reaching the logarithmic growth phase, the cells were infected with 20 MOI (multiplicity of infection) of VCS M13 helper phages (200251; Agilent Technologies) and incubated statically for 30 min at 37°C. The cells were cooled, centrifuged (at 4°C 4000 rpm for 10 min), and the pelleted cells were resuspended in glucose‐free SB medium (10 μg/mL tetracycline and 25 μg/mL chloramphenicol). After 1 h of incubation (at 30°C, 300 rpm), 50 μg/mL kanamycin (Sigma‐Aldrich) and 100 μM IPTG (Fisher Bioreagents) were added and the culture was incubated at 26°C, 300 rpm, overnight. Phage stocks were prepared by pelleting the bacterial culture and recovering the supernatant. Phages were precipitated by adding PEG/NaCl and incubated on ice for 30 min. This solution was centrifuged at 4°C, 16,000*g*, followed by additional precipitation with PEG/NaCl on ice. The supernatant was discarded after the solution was centrifuged and the phage pellet was resuspended in TSA/BSA (50 mM Tris‐HCl, 150 mM NaCl 1% BSA, pH 7.5).

### 2.3 Quantification and enrichment monitoring of phages

After a finished panning cycle, the phages were quantified with an assay where the single‐stranded DNA of the phages is measured using a lanthanide chelate complementation probe that emits a fluorescent signal proportional to the concentration of the phages. This quantification assay is better described in Lehmusvuori et al. (2012).

In addition to the previously mentioned output monitoring, the enrichment of the SSL‐binding phages was determined by a phage immunoassay after each selection round. In the immunoassay a streptavidin‐coated microtiter plate (41‐07TY; Kaivogen) was coated with the biotinylated antigens, incubated 30 min while shaking and the plate was washed four times with a washing solution (Kaivogen). Phage stocks were added in 1:2000 dilution in Red Assay Buffer (Kaivogen) and incubated for 1 h while shaking. The wells were washed four times and 125 ng/mL Eu‐N1 chelate labeled monoclonal anti‐M13 phage antibody (in Prof. Urpo Lamminmäki's laboratory, Department of Life Technologies) was added, again incubating for 1 h while shaking and washed four times. As a final step, Europium intensifier solution (Kaivogen) was added and let incubate for 10 min while shaking. Time‐resolved fluorescence was measured with a Victor plate reader (PerkinElmer). The Europium fluorescent signal is directly proportional to the amount of antigen‐bound phages.

### 2.4 Cloning, expression, and protein purification

The scFv encoding plasmids were extracted from the XL1‐blue cells from the 3rd panning round with GeneJET Plasmid Miniprep Kit (Thermo Fisher Scientific). The plasmids were digested with SfiI (Thermo Fisher Scientific) and cloned into a pLK06H plasmid vector to form a scFv‐ALP fusion protein (Huovinen et al., 2013). In addition, the plasmid contains an ampicillin resistance gene and a C‐terminal 6x histidine tag. The constructed plasmid was transformed into a One Shot Mach1‐T1 chemically competent *E. coli* following the provided instructions (C8620‐03; Invitrogen). The transformed cells were then spread on LA plates (100 μg/mL ampicillin) and incubated at 37°C overnight.

For primary screening, individual colonies were picked and transferred to 96‐well culture plates (Sarstedt) with SB medium (1% glucose, 100 μg/mL ampicillin). For each target 96 colonies, binding to either SSL1, SSL5, or SSL10, were picked. The plates were incubated at 37°C, 900 rpm, overnight. The medium was then changed to SB containing 0.05% glucose, 100 μg/mL ampicillin, and incubated at 37°C, 900 rpm until the cultures reached the log phase. The bacteria were induced with 0.2 mM IPTG for scFv‐ALP production. After an overnight incubation at 26°C the cells were lysed by adding lysis buffer (10 mg/ml Lysozyme [Sigma‐Aldrich], 10 mM MgCl_2,_ 25 U/mL benzonase [Merck]) and incubated shaking for 30 min. The cells were frozen at −70°C, thawed, and centrifuged at 4000*g*, and the supernatant was collected. Finally, the scFv‐ALP clones' ability to bind the three SSLs was determined by an ELISA‐based screen similarly as described in Section 2.3, with the exception that the SSL‐bound scFv‐ALP were detected by measuring the alkaline phosphatase activity using a p‐nitrophenyl phosphate substrate (5 mM pNPP, 50 mM Tris, 200 mM NaCl, and 10 mM MgCl_2_, pH 9) and incubated 1 h while shaking before measuring the absorbance at 405 nm.

The scFv clones displaying SSL‐binding were sequenced. Plasmid DNA was extracted from the bacteria using the Miniprep Kit (Thermo Fisher Scientific) and sequenced at Macrogen Europe (the Netherlands) with the primer WO375 (5′‐TCACACAGGAAACAGCTATGAC‐3′). The selected SSL‐binding antibody clones were produced similarly to the primary screening, only in a larger volume (50 mL) and the lysis freeze‐thaw cycles were repeated three times. After centrifugation 40 mM of imidazole was added to the lysate supernatant and applied to HisPur™ Ni‐NTA spin columns (Thermo Fisher Scientific). The lysate was purified following the provided protocol using 500 mM imidazole in phosphate‐buffered saline, pH 7.4 for elution. Protein purity was verified with sodium dodecyl sulphate‐polyacrylamide gel electrophoresis (SDS‐PAGE) and the protein concentration was determined with NanoDrop (Thermo Fisher Scientific) at 280 nm. The scFv‐ALP antibodies were stored at 4°C.

### 2.5 Fluorogenic peptide conversion MMP activity assay

To determine if the SSL1 and SSL5 recognizing antibodies were able to inhibit their targets, we screened the scFvs in a fluorogenic peptide conversion MMP9 activity assay. Here we investigated if the antibodies could block the SSLs inhibitory effect and maintain MMP9 proteolytic activity by measuring the fluorescence emitted by a fluorogenic peptide that the MMP9 cleaves (Koymans et al., 2016). Both SSL1‐ and SSL5‐binding antibodies were initially tested at a higher concentration of 50 μg/mL (data not shown) and the scFvs showing inhibition of the SSLs were further tested in the same assay using a concentration series.

Pro‐MMP9 (R&D Systems) was diluted to a concentration of 100 μg/mL and activated with trypsin (10 μg/mL) in assay buffer (50 mM Tris, 10 mM CaCl_2_, 150 mM NaCl, 0.05% Brij‐35, pH 7.5) incubating at 37°C for 2 h. Trypsin was inactivated with 100 μg/mL α−1 antitrypsin (Sigma‐Aldrich). The SSLs (1 μg/mL SSL1 or 0.5 μg/mL SSL5) were preincubated with a concentration series (final concentrations ranging between 50 and 0.16 μg/mL) of scFv in a clear microtiter plate (442404; Thermo Fisher Scientific) in a total volume of 25 μL for 30 min. Afterward, the activated MMP9 was added to the microtiter plate to a final concentration of 1.0 μg/mL and incubated at room temperature (RT) for 30 min. Finally, 50 μL of Mca‐KPLGL‐Dpa‐AR‐NH2 fluorogenic peptide substrate IX (R&D Systems) was added, making the total volume up to 100 μL. Fluorescence intensity was measured at excitation and emission wavelengths of 320 and 405 nm, respectively, for 40 min (100 cycles, 24 s cycle time) with a Clariostar microplate reader (BMG Labtech). The MMP9 activity was determined by calculating the area under the curve after taking a blank measurement into account.

### 2.6 MMP9‐degraded collagen and gel chromatography

To further confirm the SSL inhibition, the scFv function was determined by MMP9 degraded collagen that was visualized on an SDS‐PAGE. Pro‐MMP9 was activated similarly to the fluorogenic peptide assay except that the trypsin inactivation was replaced by a trypsin inhibition mixture (10 mM Pefabloc [Sigma‐Aldrich], 3,5 mg/mL STBI, 100 mg/mL Aprotinin [Sigma‐Aldrich]) incubating for 30 min at RT. SSL1 (20 μg/mL) and the scFvs were preincubated in a total volume of 6 μL for 30 min at RT before the addition of MMP9 (final concentration of 5 μg/mL). Finally, collagen type I (0.5 mg/mL) (human; Sigma‐Aldrich) was added and incubated at 37°C overnight. The following day 2× sample buffer (with 50 mg/mL DTT [Sigma‐Aldrich]) was added, and the samples were run on a NuPAGE 4‐12% Bis‐Tris Gel (Invitrogen) at 180 V. The gel was stained with Instant Blue protein stain (Expedeon).

### 2.7 PSGL‐1 competitive binding assay

A PSGL‐1 competitive binding assay was carried out to determine the ability of scFvs to interfere with SSL5 binding to the PSGL‐1 receptor and allow the interaction with the receptor's natural ligand, P‐selectin (Bestebroer et al., 2007). Initially, SSL5 and P‐selectin concentrations were optimized to determine the concentrations showing the clearest difference between the SSL‐inhibited and free PSGL‐1. The antibodies (50 μg/mL) were preincubated with SSL5 (3 μg/mL) at RT for 15 min. Neutrophils (5 × 10^6^ cells/mL) were added to this mixture and incubated on ice for another 15 min. P‐selectin‐Fc chimera (0.3 μg/mL [R&D System]) was allowed to bind to PSGL‐1 on the neutrophil surface for 30 min on ice. The cells were washed with an assay buffer containing RPMI 1640 supplemented with 0.05% HSA (Gibco). To visualize the P‐selectin‐Fc bound to the PSGL‐1 receptor, Fc binding Donkey‐anti‐human‐IgG(H + L)‐APC (diluted in assay buffer in a 1:400 ratio [Jackson Immuno Research]) was added and incubated on ice for 30 min. Finally, the cells were washed with assay buffer and the APC fluorescent signal was measured with a FACSVerse flow cytometer (BD Bioscience). The neutrophils were selected by forward and side scatter gating.

### 2.8 IgG1‐mediated phagocytosis assay

An IgG1‐mediated phagocytosis assay was carried out to determine if the SSL10‐specific scFvs could block SSL10 and enable a normal Fc‐mediated bacterial uptake by neutrophils (Itoh et al., 2010b). All reagents used in this assay were diluted in assay buffer (RPMI‐1640 supplemented with 0.05% HSA). The scFvs (5 µL; final concentration 50 μg/mL) were preincubated with 5 µL SSL10 in a 96‐well plate for 15 min at 37°C shaking at 750 rpm. Afterward, 10 µL human anti‐WTA IgG1 was added to the wells and further incubated in the same conditions as previously. In the following step 20 µL 3.75 × 10^7^ b/mL *S*. *aureus* (GFP‐labeled Wood 46) was added and incubated for another 15 min. Finally, 10 µL polymorphonuclear leukocytes (PMN) (7.5 × 10^6^ c/mL) were added and allowed to phagocytize the IgG1 opsonized bacteria shaking at 37°C for 15 min. Afterward, the cells were fixed with paraformaldehyde and stored at 4°C. The following day, the cells were analyzed by flow cytometry (FACSVerse; BD Biosciences).

### 2.9 Structural model of scFv‐93

Since we already had determined the sequences of the scFvs, it was possible to construct a homology model of the inhibiting scFv‐93. A suitable structural template was found through a sequence similarity search performed with BLAST (Basic Local Alignment Search Tool) (Altschul et al., 1990) against Protein Data Bank (PDB) (Berman, 2000). Two scFv antibody structures were chosen as modeling templates: A mouse scFv (PDB ID: 2KH2), (Wilkinson et al., 2009) and a human scFv (PDB ID: 6TCS), (Mitropoulou et al., 2020) with e‐values of 2 × 10^−115^ and 6 × 10^−108^, respectively. A multiple sequence alignment was created of scFv‐93 and the two template scFvs were created with the Clustal Omega online tool (Sievers et al., 2011). The program MODELLER (Šali & Blundell, 1993) was used to build 10 3D models and the model with the lowest value according to the MODELLER DOPE (Discrete Optimized Protein Energy) score was chosen for further analysis.

### 2.10 Protein–protein docking

To create a model of SSL1 and scFv‐93 as a complex, protein–protein docking analysis was carried out with HADDOCK (High Ambiguity Driven protein–protein DOCKing) web server 2.4 (Van Zundert et al., 2016). The crystal structure for SSL1 was retrieved from the PDB (PDB ID: 4O1N, chain A). Even though there is no direct experimental data on the binding interface of the scFv‐SSL1 complex, the SSL1 inhibiting function of the antibody reveals a possible binding site being at the same interface that binds to MMP9. Since there is no experimental data on the SSL1‐MMP9 interaction, we used the SSL5‐MMP9 interface as a starting point Kohno et al. (2018). Two complex models were created by docking assigning either the SSL1 C‐terminal α4β9 domain (^138^LDYRLRERAIKQHGLYSNGLKQGQITITMNDGT^170)^ or the N‐terminal β1‐3 (^25^PILERKNVTGFKYTDEGKHYLEVTVGQQHSRITLLG^60^) as active residues (binding site residues) mimicking the SSL5 binding sites to MMP9 as reported by Kohno et al. (2018). The H2 and the H3 loops of the antibody were assigned as active residues on both complexes.

### 2.11 Molecular dynamics simulations

To further analyze the built complexes, three 100‐ns simulations were performed with the Desmond Molecular Dynamics program as implemented in Schrödinger's Maestro Molecular Modeling Suite (Schrödinger Release 2020.4; Schrödinger, LLC, New York, 2020) (Bowers, 2006). Originally, the MD parameters were adopted from Marimuthu et al. (2021). In short, the complexes were preprocessed using the Protein Preparation Wizard (Schrödinger Release 2020.4: Preparation Wizard; Schrödinger, LLC, New York, NY, 2020) in the Maestro molecular modeling software by adding hydrogens and charges using the OPLS4e force field, which was used throughout this experiment (Harder et al., 2016; Jorgensen & Tirado‐Rives, 1988; Jorgensen et al., 1996; Madhavi Sastry et al., 2013; Shivakumar et al., 2010). This was followed by an optimization of the hydrogen bonding networks and finally, a restrained minimization was performed (including hydrogens and heavy atoms up to RMSD of 0.3 Å). To neutralize the system, two Cl^−^ ions were added and extra Na^+^/Cl^−^ ions were included to obtain a salt concentration of 0.15 M. Before the production run, a multistage equilibration protocol (as detailed in Marimuthu et al., 2021) was carried out for the system. The production simulation was carried out at 300 K using the TIP3P explicit solvent model (Jorgensen et al., 1983) in an orthorhombic box extending 10 Å from the surface of the solute. A time step of 2 fs was used, and altogether a 100‐ns simulation trajectory was recorded at intervals of 100 ps that resulted in 1000 snapshots (frames) by the end of the simulation.

The estimated binding free energy of the simulated SSL1‐scFv‐93 complexes was calculated for 10 snapshots from the trajectory starting from 50 ns onward every 5 ns using Schrödinger's Prime/MM‐GBSA (molecular mechanics‐generalized Born surface area) method in Maestro. The VSGB solvation model (Li et al., 2011) and the OPLS4e force field were used for the MM‐GBSA analysis. Parameters such as protein root‐mean‐square deviation (RMSD) and protein root‐mean‐square fluctuation (RMSF) were also investigated from the MD trajectories. In addition, the intermolecular residue contacts at the protein–protein interface were analyzed from the last 50 ns of the simulation.

## 3 RESULTS

### 3.1 Phage enrichment and detection of binding scFvs

Two synthetic scFv phage libraries, scFvM and scFvP with different binding sites and diversities designed especially for haptens and proteins, respectively (Huovinen et al., 2013) were phage display selected for three rounds against SSL1, SSL5, and SSL10. Phage enrichment and specificity towards the three targets were monitored by two methods: output titrations and a phage immunoassay. As the phages were enriched toward their targets, the output/input ratio increased and the percentage of the background signal decreased (Table 1). The enrichment was also seen as an increase in the signal in the immunoassay that measured phage binding to the target antigen.

**Table 1:** Enrichment of phages during panning against SSL1, SSL5, and SSL10.^a^

	1st round	2nd round	3rd round
All targets			
Phage input (cfu)	5.0 × 10^12^	1.0 × 10^11^	1.0 × 10^11^
SSL1			
Phage output (cfu)	2.2 × 10^7^	4.2 × 10^7^	7.4 × 10^7^
Phage recovery (% input)	0.00044	0.042	0.074
Background (%)	–	1.0	0.0020
SSL5			
Phage output (cfu)	1.3 × 10^7^	4.0 × 10^6^	7.1 × 10^7^
Phage recovery (% input)	0.00025	0.0040	0.071
Background (%)	–	16.2	0.0081
SSL10			
Phage output (cfu)	4.3 × 10^7^	7.5 × 10^6^	8.2 × 10^7^
Phage recovery (% input)	0.00086	0.0075	0.082
Background (%)	–	5.5	0.015

^a^ Phage recovery was calculated by dividing the number of phages collected (output) from each target antigen by the number of phages used in panning (input). The total number of eluted phages was determined by plating serially diluted infected cultures. The number of phages used for the first panning round was 5.0 × 10^12^ and for the second and third rounds was 1.0 × 10^11,^ regardless of the antigen. The background was calculated as the percentage of the collected phages that presented unspecific binding. Abbreviation: cfu, colony forming units.

**Figure 2:**
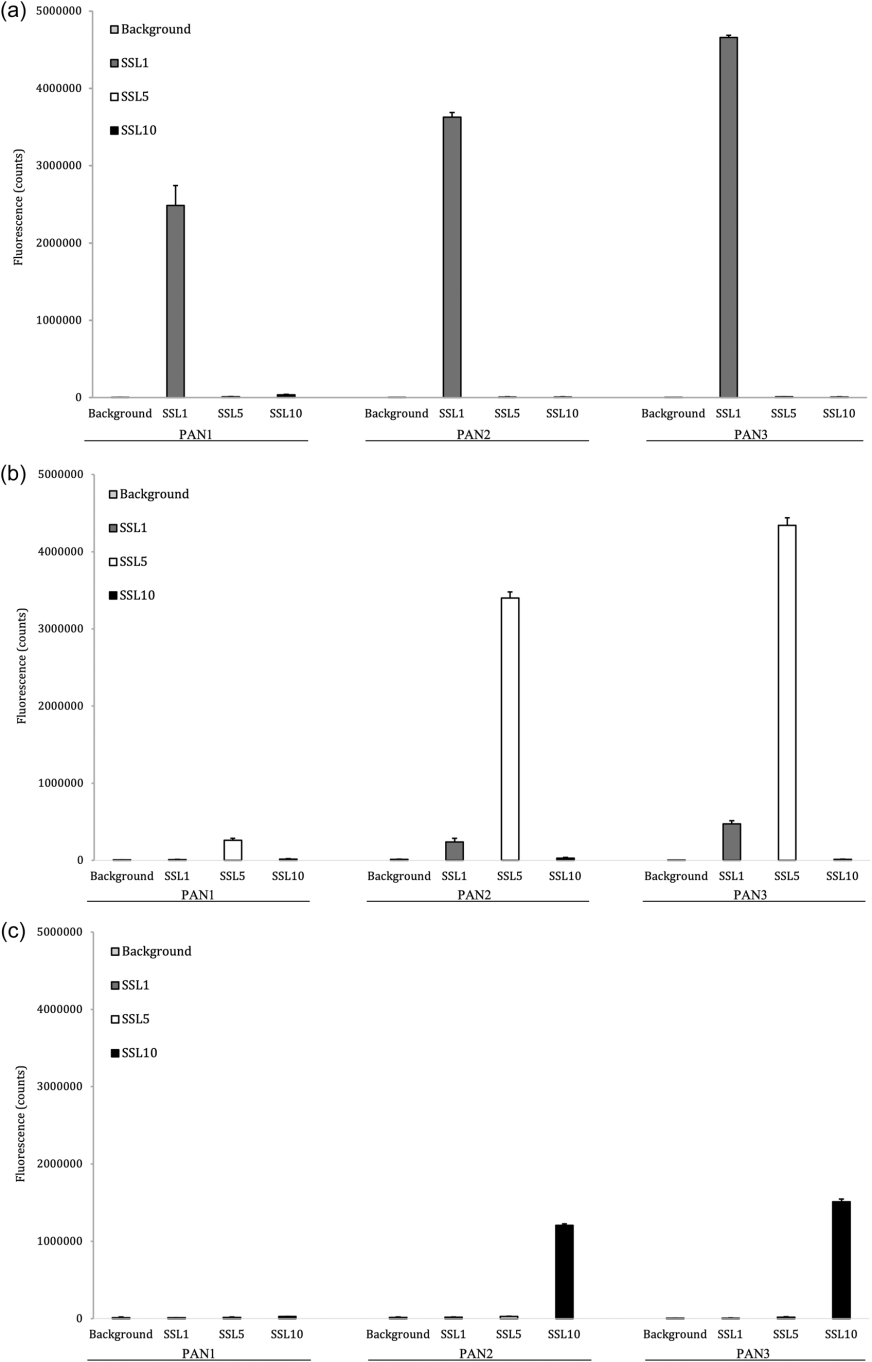
(a–c) Enrichment of the phage antibody library against. (a) SSL1, (b) SSL5, or (c) SSL10 determined by phage immunoassay after each panning round. The bars represent an average of triplicate fluorescent signals obtained and their respective standard deviations.

In all three panning experiments (SSL1, SSL5, and SSL10 as targets) the enriched phage libraries became more selective towards their targets (Figure 2). Already after the first round the SSL1‐phage stock showed a clear enrichment towards its intended target, which could be observed from the phage immunoassay (Figure 2a) and the low background when monitoring the phage‐infected cultures (Table 1). Overall, the phages panned against SSL1 enriched most efficiently with a 170‐fold enrichment of phages between the first and the third panning round (Table 1). The selections against SSL5 and SSL10 produced a significant enrichment first after the second panning round. This is also clear from the higher background percentages after the first panning rounds (Table 1). After the third and final panning round, the phages of the SSL5‐ and SSL10‐selection had reached a 283‐ and 96‐fold enrichment, respectively.

### 3.2 Identifying target‐binding clones

**Figure 3:**
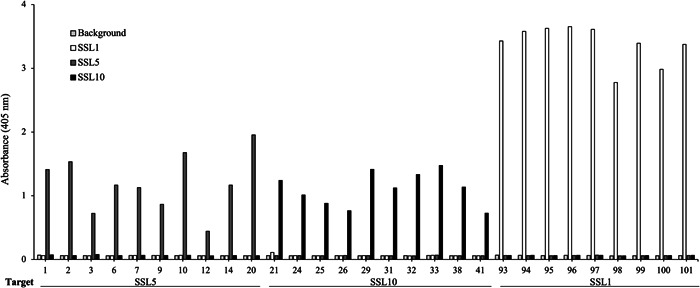
Binding profiles for the 29 SSL‐binding scFv‐ALP (alkaline phosphatase fusion scFv) chosen for further activity testing. The binding of the scFvs to the biotinylated antigens was detected through the fusion ALP activity. scFv‐ALP, alkaline phosphatase fusion proteins.

**Figure A1:**
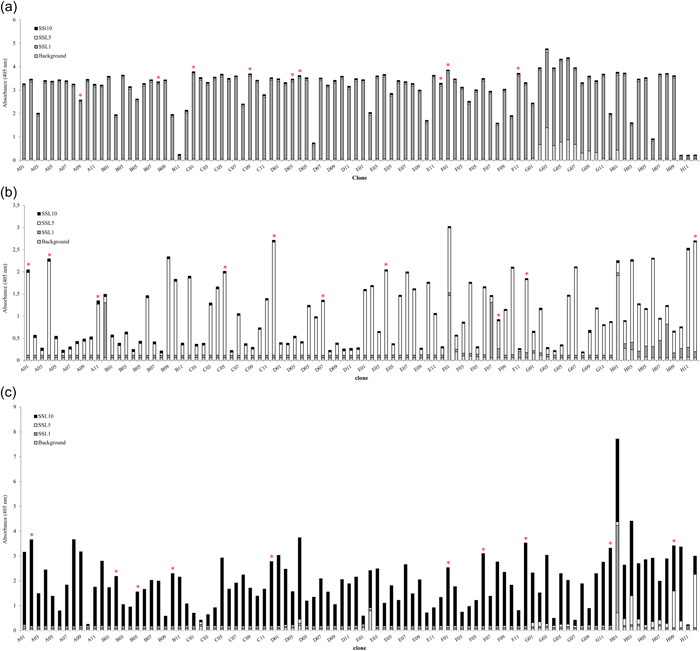
(a–c) Screening immunoassay results for SSL‐binding scFv‐ALP clones after three rounds of panning. The binding of the scFvs to the biotinylated antigens was detected through the fusion ALP activity. The clones selected for larger‐scale production and further activity testing are denoted with a red asterisk. ALP, alkaline phosphatase; scFvs, selective single chain variable fragment.

After three rounds of panning against SSL1, SSL5, and SSL10, 96 individual clones were picked up for each target and produced as alkaline phosphatase fusion proteins (scFv‐ALP) on microtiter plate cultures and screened by ELISA to find SSL‐binding clones. The ELISA screen was conducted with antibodies directly from the plate cultures. Altogether 3 × 96 clones were included in the primary screen (Figure A1). Generally, the antibodies panned against SSL1 showed a higher binding signal towards their target than SSL5 and SSL10 panned antibodies, which is also evident from Figure 3. It should be noted that it is possible that the higher binding signal could be due to factors such as the producibility of certain clones. In total 52 SSL‐binding clones were chosen for sequencing, and out of these 44 unique sequences were obtained. The two phage libraries had diversity in the antibody CDR (complementarity determining region) loops L1, L3, H1, H2, and H3 (L for the light chain and H for the heavy chain) (Huovinen et al., 2013). All the sequenced SSL1‐binding clones had a seven amino acid long CDR‐H3 loop, two pairs of them being identical. The CDR‐H3 loops of SSL5 and SSL10‐binding antibodies varied more with lengths from 6 to 18 amino acids. Based on the CDR‐H3 sequences and the binding profiles on the ELISA‐binding screen (ABS 405 nm cutoff >0.4) 29 clones were chosen for a larger scale production and purified to be studied further in functional assays to determine their possible inhibitory activity against the SSLs (Figure 3).

### 3.3 ScFvs inhibit SSL1 and SSL5 and maintain peptide cleavage by MMP9

To determine if the scFvs produced against SSL1 and SSL5 were able to inhibit their targets, we screened the scFvs in a fluorogenic peptide conversion MMP9 activity assay. The SSLs were pretreated with antibodies, and the inhibitory potential of the SSLs was assessed by measuring MMP9 activity through a fluorogenic peptide substrate that emits fluorescence after MMP9 cleavage of the quenching group. The SSL‐binding antibodies were initially tested at a higher concentration of 50 μg/mL (660 nM) (data not shown) and the scFvs showing inhibition of the SSLs were further tested in a concentration series.

**Figure 4:**
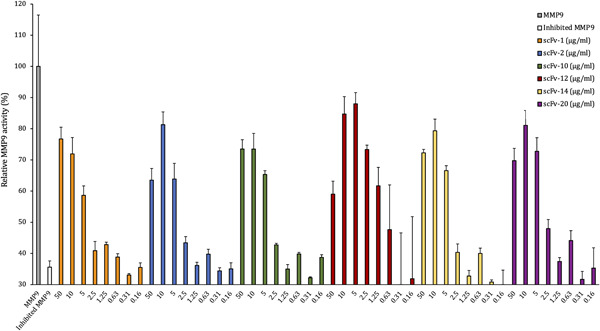
Effect of SSL5‐binding scFvs on relative MMP9 activity after inhibition by 0.125 μg/mL SSL5. The gray bar represents the enzymatic activity of MMP9 with no SSL present and the white bar its activity after the addition of SSL5. The rest of the bars represent MMP9 activity after the addition of SSL5 that was pretreated with a concentration range (in μg/mL) of SSL5‐binding scFvs. MMP9, matrix metallopeptidase 9; scFvs, selective single chain variable fragment.

Out of the 10 initially tested SSL5‐binding antibodies six could inhibit SSL5: 60%–80% of MMP9 activity was recovered with the initial high scFv concentration (scFvs 50 μg/mL/660 nM and SSL5 0.125 μg/mL/4.5 nM, molar ratio of 147:1). For scFv‐2, −12, −14 and −20 the lower 10 μg/mL concentration was even more effective in inhibiting SSL5 (molar ratio 29:1 between the scFvs and SSL5, Figure 4). From the concentration of 5 μg/mL downwards the scFv gradually lost its recovering effect on MMP9.

**Figure 5:**
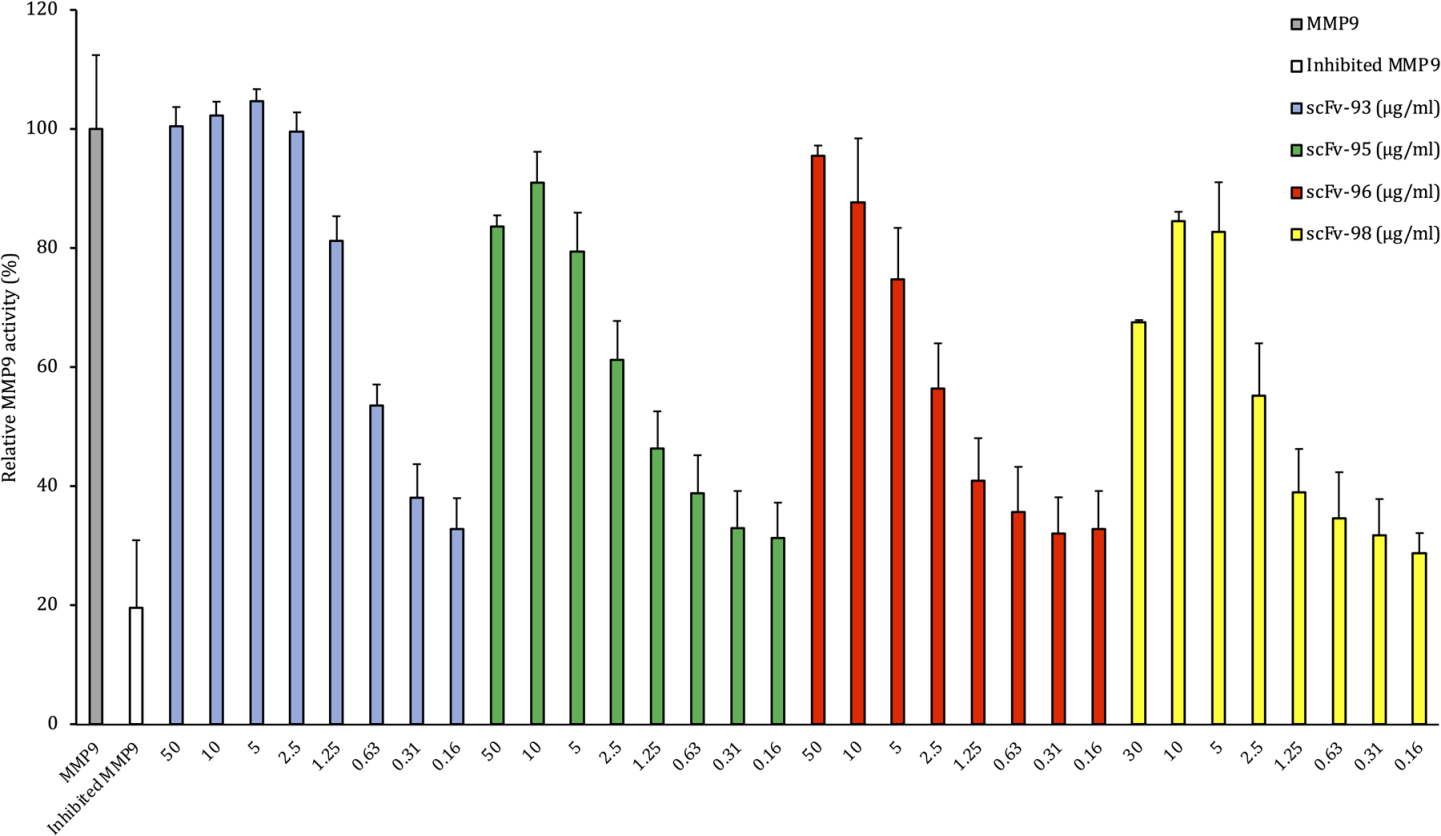
Relative MMP9 activity after SSL1 inhibition by 0.25 μg/mL SSL1. The gray bar represents MMP9 enzymatic activity with no SSL1 present and the white bar the activity after the addition of SSL1. The rest of the bars represent MMP9 activity after the addition of SSL1 that was pretreated with a concentration range (in μg/mL) of SSL1‐binding scFvs. MMP9, matrix metallopeptidase 9; scFvs, selective single chain variable fragment.

Four SSL1‐binding antibodies out of the 10 tested were able to inhibit 0.25 μg/mL SSL1 (9.6 nM) at the initial high concentration (660 nM, for scFv‐98 the highest concentration was 390 nM) recovering MMP9 activity between 70% and 100% (Figure 5). These four antibodies were able to inhibit SSL1 inhibition in a concentration‐dependent manner. scFv‐93 was the best‐performing antibody out of the four: even at the concentration of 1.25 μg/mL (16 nM) it was able to inhibit SSL1 and recover MMP9 activity up to 80%. For the rest of the scFvs, the inhibitory activity gradually disappeared using concentrations lower than 10 μg/mL (130 nM).

### 3.4 ScFv‐93 inhibits SSL1 and maintains MMP9 collagen degradation

**Figure 6:**
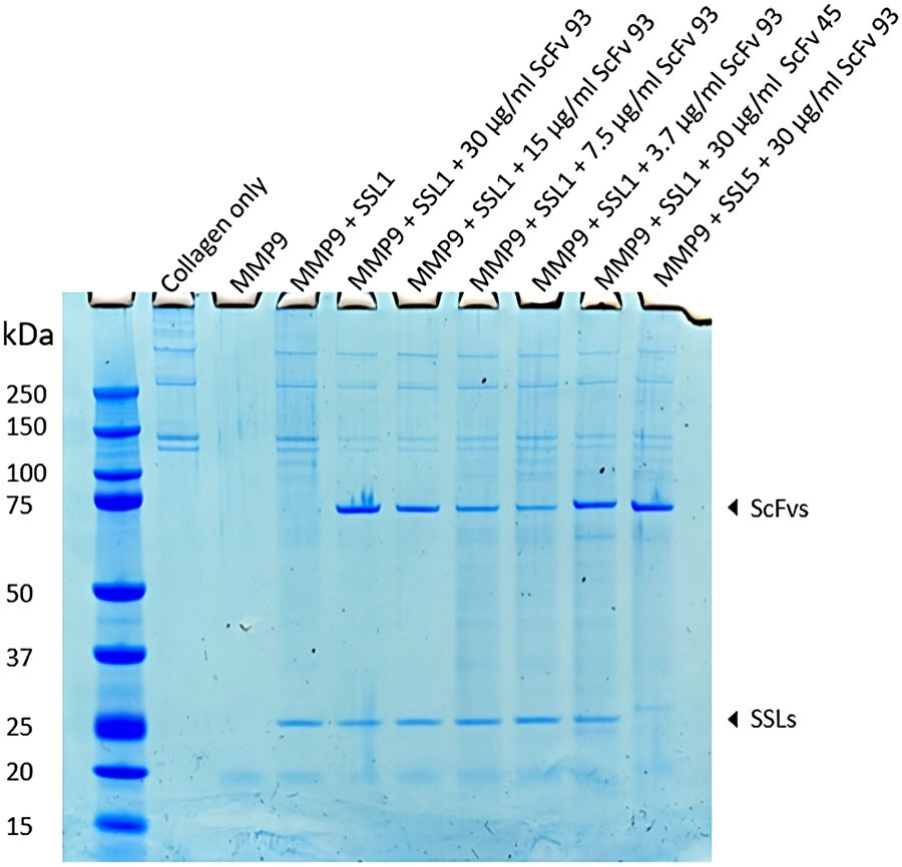
MMP9‐mediated collagen degradation visualized on an SDS‐PAGE. All the wells contain collagen. Activated MMP9 and MMP9 inhibited with SSL1 were used as positive and negative controls, respectively. Noninhibiting scFv‐45‐ALP and SSL5 that scFv‐93‐ALP does not recognize were used as additional controls. scFv‐93‐ALP was added in a concentration series from 3.7 to 30 μg/mL. MMP9, matrix metallopeptidase 9; scFvs, selective single chain variable fragment; SDS‐PAGE, sodium dodecyl sulphate‐polyacrylamide gel electrophoresis.

The scFv‐93 was further examined in a collagen degradation assay visualized on SDS‐PAGE. As seen from Figure 6 collagen alone accounts for two bands around 150 kDa and two bands above 250 kDa. These four bands disappear completely when MMP9 is added and reappear when MMP9 is inhibited with SSL1. ScFv‐93 was used to inhibit 3 μg/mL (120 nM) SSL1 in a dilution series from 30 μg/mL (400 nM) to 3.75 μg/mL (50 nM). As additional controls, a noninhibiting scFv‐45 and SSL5 that scFv‐93 does not recognize were used. scFv‐45 was observed to bind to SSL1 in the ELISA but not to inhibit it in the fluorogenic peptide conversion MMP activity assay. In this assay, we could not show as clear SSL1 inhibition by scFv‐93 as in the fluorogenic peptide assay. However, the bands for collagen in the lanes with added scFv‐93 appear fainter compared to the lane with SSL1 and MMP9 without scFv, indicating a partial recovery of MMP9 enzymatic activity. The collagen bands increase in intensity as the scFv‐93 concentration is reduced, and at 50 nM scFv‐93, no inhibition of SSL1 can be detected anymore. The gel also confirms that scFv‐93 does not inhibit SSL5.

### 3.5 Gel filtration chromatogram reveals an scFv‐93‐SSL1 complex formation

**Figure 7:**
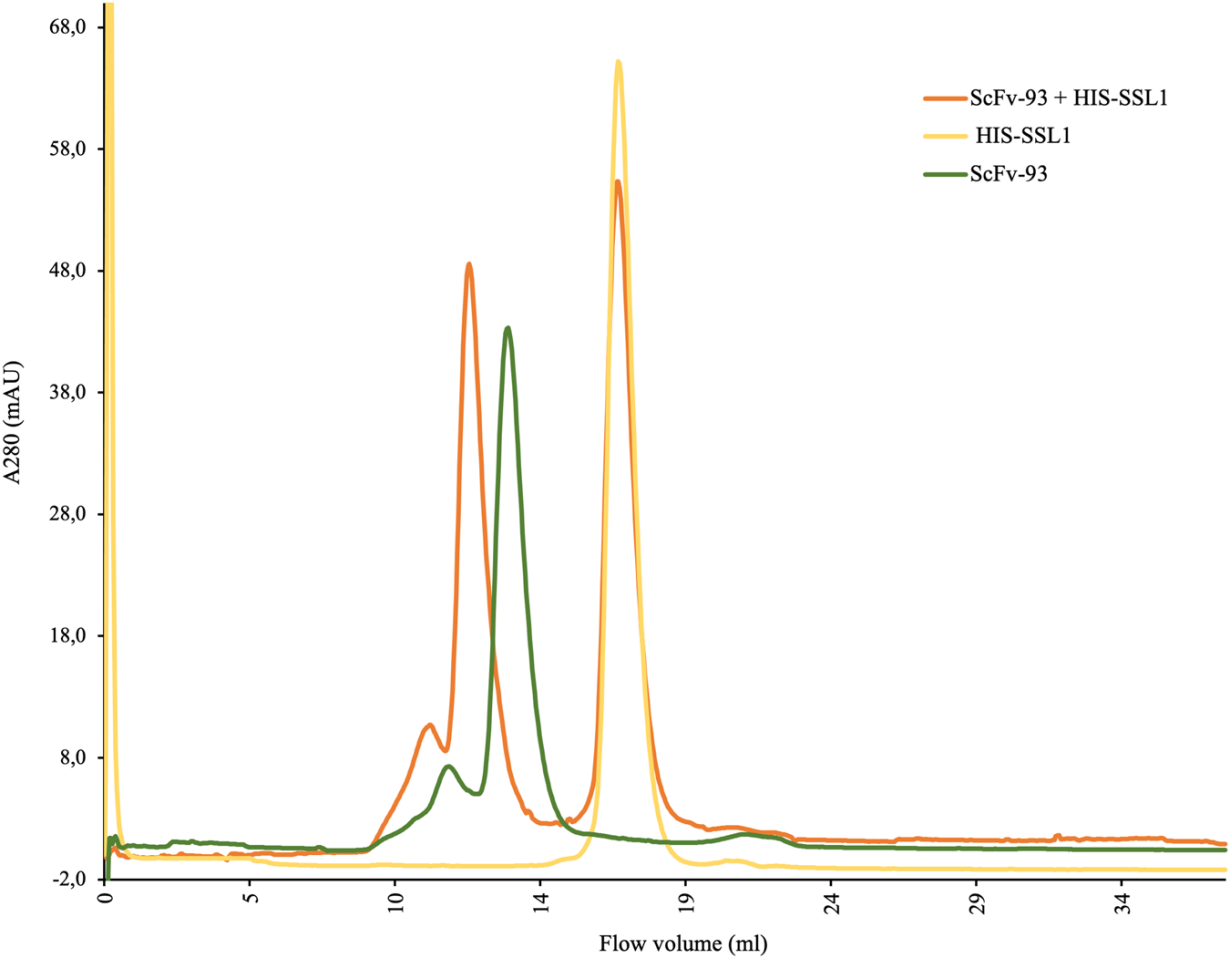
Gel filtration chromatogram. 280 µg/mL scFv‐93‐ALP was preincubated with 500 µg/mL HIS‐SSL1 in a total volume of 500 µL for 30 min at RT before running on a gel filtration column. For comparison, 280 µg/mL scFv‐93‐ALP and 500 µg/mL HIS‐SSL1 were run separately. ALP, alkaline phosphatase; RT, room temperature.

The scFv‐93 binding to SSL1 was further confirmed by gel filtration chromatography (size‐exclusion chromatography) using a Superdex 200 10/300 GL column (Figure 7). First, only scFv‐93 and SSL1 were applied to the gel filtration column in separate runs. When scFv‐93 and SSL1 were incubated together, a new faster eluting peak, indicating the formation of a complex, was obtained, as the concentration of SSL1 was fivefold higher on a molar basis as compared to scFv‐93, scFv‐93 was fully complexed with SSL1, leaving only uncomplexed SSL1.

### 3.6 SSL5‐binding scFvs cannot inhibit SSL5 binding to PSGL‐1

**Figure 8:**
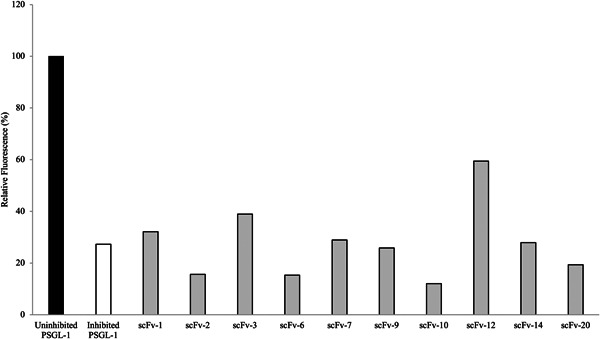
ScFvs' effect on P‐selectin binding to PSGL‐1 on human neutrophils in the presence of SSL5. P‐selectin binding to PSGL‐1 was measured after the addition of SSL5 (1.0 μg/mL) and scFvs (50 μg/mL). The control of uninhibited PSGL‐1 was measured and the signals for the rest of the measurements were adjusted in relation to its fluorescent signal. A low fluorescent signal indicates a low level of PSGL‐1 bound P‐selectin. scFvs, selective single chain variable fragment.

To further test the SSL5‐binding scFvs, the antibodies were analyzed for their ability to inhibit SSL5 binding to PSGL‐1 and thereby allow normal P‐selectin binding (Bestebroer et al., 2007). In this PSGL‐1 competitive binding assay, we used 1.0 μg/mL (36 nM) of SSL5 which was able to inhibit 72.7% of P‐selectin binding to PSGL‐1 (Figure 8). When the SSL5‐binding antibodies were added, no significant improvement in P‐selectin binding could be observed. ScFv‐12 showed a modest improvement of 30% in the P‐selectin binding compared to the inhibited PSGL‐1, whereas scFv‐3 showed only a minor improvement of 12%. The scFvs were added in a high concentration of 50 μg/mL (670 nM) which is more than 18 times the amount of SSL5 used.

### 3.7 SSL10‐binding scFvs cannot block SSL10 binding to IgG1

**Figure A2:**
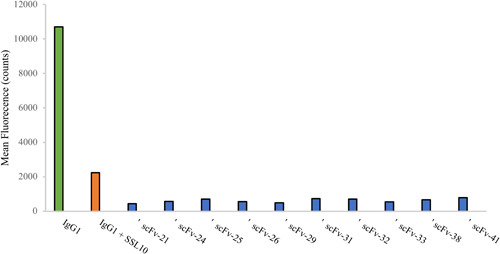
IgG1 mediated phagocytosis. Uninhibited IgG1 is shown as a green bar, SSL10 inhibited IgG1 is shown as an orange bar and the scFv pretreated SSL10 with IgG1 are shown as blue bars. No SSL10 inhibition of the antibody fragments could be detected.

The scFvs panned against SSL10 were tested in a phagocytosis assay where we used the SSL10‐IgG1 interaction as a basis (Itoh et al., 2010b). Our goal was to inhibit SSL10 with our scFv antibodies so that neutrophils would recognize and phagocytize the IgG1‐opsonized *S*. *aureus* normally. SSL10 was pretreated with the scFvs before the addition of human IgG1. We used fluorescent *S*. *aureus* to be able to measure the neutrophil phagocytized bacteria by flow cytometry. However, we could not show that any of the 14 tested scFvs could block SSL10 from binding to IgG1 (Figure A2).

### 3.8 Modeling and molecular dynamics simulations of the scFv‐93‐SSL1 complex

**Figure 9:**
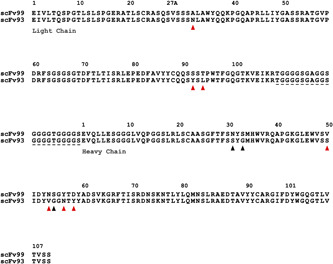
A pairwise alignment of the SSL1‐binding scFv‐99 and SSL1‐binding and inhibiting scFv‐93. Black arrows indicate conservative residue variation and red arrows nonconservative variation. Most of the variation between these antibodies is located in the CDR‐H2 loop (from residue 50 to 65; Kabat numbering; Kabat et al., 1991). The linker joining the variable chains together is marked with a dashed line. Sequence alignment was conducted with Clustal Omega (1.2.4) (Figure A4) and modified with the ESPript online tool (Madeira et al., 2019; Robert & Gouet, 2014).

As the scFv antibodies were isolated from a synthetic antibody library that uses a single framework, other than the diversified CDR‐L1, ‐L3, ‐H1, ‐H2, and ‐H3 loops, the scFv sequences were conserved, and the antibody sequence identities remained above 90% in all the sequenced scFv clones. One SSL1‐binding antibody, scFv‐99, was found to share 96.1% identity with the SSL1‐inhibiting scFv‐93 (Figure 9). There is a 10‐residue difference between the two antibodies, out of which seven residues vary nonconservatively (Figure 9). Most of the residue variation resides in the CDR‐H2 loop while the CDR‐H3 loops, often the main contributor for antibody specificity, are identical. Since only the scFv‐93 can block SSL1 inhibition of MMP9, it is possible to conclude that these 10 residues include also the key amino acids that contribute towards the SSL1 inhibiting function.

A homology model of the scFv‐93 was built with MODELLER without the ALP‐fusion. The SSL5‐MMP9 interacting domains have been studied previously (Kohno et al., 2018) and SSL1 likely has a similar binding mode to MMP9 as SSL5. Thus, SSL1 is likely to bind to MMP9 with its N‐terminal β1‐3 (^25^PILERKNVTGFKYTDEGKHYLEVTVGQQHSRITLLG^60^) domain or the C‐terminal α4β9 domain (^138^LDYRLRERAIKQHGLYSNGLKQGQITITMNDGT^170^) (see Figure 1). We hypothesize that scFv can block either one of these SSL1 sites. These putative binding sites were used as a basis when creating a complex model of SSL1 and inhibiting scFv‐93 by protein–protein docking with HADDOCK. The CDR‐H2 and the CDR‐H3 loops of the antibody were assigned as active residues on both complexes. The stability of the predicted complexes was further analyzed by subjecting them to 100‐ns molecular dynamics (MD) simulations.

In both MD‐simulated models, the RMSD increased for the initial 20–30 ns of the simulation and reached a steady state at the last 50–70 ns. Overall, the MD simulation showed good dynamic stability for both complexes. However, the further binding free energy calculations revealed more favorable binding towards the SSL1 N‐terminal β1‐3 site (Table 2). The complex model with the N‐terminal site gave an average binding free energy (Δ*G*) value over three times lower than the model with the C‐terminal site (the more negative a value, the more favorable a binding energy). In addition, some snapshots from the C‐terminal binding model trajectory even gave a positive Δ*G* indicating a suboptimal interaction between the antibody and the SSL1 α4β9 domain.

**Table 2:** Binding free energies (Δ*G* in kcal/mol) were estimated for both scFv‐SSL1 complexes from the last 50 ns of the MD trajectory at the time interval of 5 ns, that is, for 10 snapshot frames.

	ΔG Average (kcal/mol)	SD	Range
scFv‐93–SSL1 (β1‐3 binding interface)	−102.90	22.56	−150.01 to −58.78
scFv‐93–SSL1 (α4β9 binding interface)	−32.62	35.92	−79.59 to 14.51

*Note*: The Δ*G* values are combined averages of three replica MD simulations.

Abbreviation: MD, molecular dynamics.

**Figure 10:**
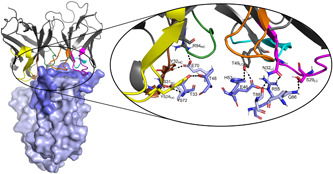
A predicted binding complex of SSL1 with scFv‐93. A crystal structure of SSL1 (PDB ID: 4O1N) was docked to the homology model of scFv‐93 and the docking complex was subjected to a 100‐ns MD simulation. The last frame of the simulation is presented here. SSL1 is presented as a light blue surface/sticks and the N‐terminal β1‐3 domain residues are colored slate blue. The CDR‐loops of scFv‐93 are colored as follows: L1 magenta, L2 cyan, L3 orange, H1 brown, H2 yellow, and H3 green. Interacting residues are labeled and presented as sticks (right). Polar contacts are shown as black dashed lines. MD, molecular dynamics.

The last MD frame of the model of the SSL1‐scFv‐93 complex with the SSL1 N‐terminal β1‐3 binding interface is shown in Figure 10. A figure of the modeled complex with the SSL1 C‐terminal binding interface can be found in the Appendix (Figure A5). Both models suggest that the interacting sites in the antibody are evenly distributed among the CDR loops. The relatively short CDR‐H3 loop of scFv‐93 creates a groove in the middle of the antigen binding domain allowing the other CDR loops closer contact with the antigen. This phenomenon has also been previously observed by Tsuchiya and Mizuguchi (2016).

Analyzing the interactions during the MD simulation revealed a couple of more prominent interactions that occurred throughout the trajectory (Figure A6). Interestingly, some of these interacting residues are among the 10 residues that differed between the scFv‐93 and scFv‐99 (Figure 8). One of these is Ser31 in the scFv‐93 H1 loop that forms an H‐bond with SSL1 Thr33 throughout the simulation. In scFv‐99, Ser31 is (conservatively) replaced by asparagine. In addition, Asn32 in the scFv‐93 L1 loop has continuous polar contact with SSL1 Arg55 during the simulation. The corresponding residue in scFv‐99 is an alanine (thus, lacking the polar interaction). However, many of the residues in the more prominent interactions are the same in the two antibodies: Tyr49 adjacent to the L2 loop forming an H‐bond with the Glu46 at the SSL1 β1‐3 site and Tyr32 and Arg94 in the H1 loop both simultaneously interacting with SSL1 Glu70.

## 4 DISCUSSION

This study reports on the identification of phage‐displayed scFv antibodies that recognize SSL1, SSL5, and SSL10. The immune assays revealed a strong and specific binding of phages against SSL1 already after the first panning round, whereas phages against SSL5 and SSL10 had more unspecific background binding during the first rounds. The signals in the ELISA screen continued this same trend, the SSL1‐binding clones gave a significantly stronger signal compared to the clones binding the other two targets. The varying results between panning targets might be purely coincidental (phage library suitability) but might also be due to suboptimal protein target concentration or biotinylation. As previously mentioned, it is also possible that the producibility of certain clones affected the intensities of the binding signals.

In the functional analysis, we could identify antibodies that inhibited some SSL5 functions at high concentrations and four antibodies that showed SSL1 inhibition in a concentration‐dependent manner. As expected, the scFvs blocking SSL5 in the MMP9 fluorescent peptide conversion assay had no overlap in the PSGL‐1 competitive binding assay. This is because the SSL5 can bind both PSGL‐1 and MMP9 simultaneously creating a triple complex, indicating that SSL5 has a different binding mode for the respective targets (Koymans et al., 2016). Out of the four SSL1 inhibiting antibodies one, scFv‐93, was able to completely block SSL1 inhibition against MMP9 and fully restore MMP9 enzymatic activity. At the concentration (16 nM) scFv‐93 was able to inhibit 9.6 nM SSL1 (molar ratio of 2:1) and recover MMP9 activity up to 80%. Previously Oku et al. (2018) also identified a monoclonal antibody specific for SSL5 using a mouse hybridoma clone. The antibody recognizes SSL5 without cross‐reactivity with other SSLs. It was shown to bind to the C‐terminal β‐grasp motif of SSL5, but it does not interfere with the binding of SSL5 to MMP9.

The observed lack of inhibition against SSL10 by the scFvs and a low inhibition against the two other SSL targets could be due to their low affinity along with a suboptimal binding site. Determining the affinity of the scFvs could shed light on this but since the purpose of this study was to identify scFv binders that are functionally capable of inhibiting SSLs, measuring the affinities of nonactives would not provide relevant information. Previous studies on these scFv phage libraries have found binders to different targets with affinities ranging from 2 to 6 nM, which should be sufficient for inhibition (Huovinen et al., 2013). However, it would be interesting to measure the affinities of scFv‐93 and scFv‐99 to SSL1 in the future, as they may bind to the same site but the scFv‐99 just with a lower affinity. These scFvs have identical CDR‐H3, which is often the key specificity‐determining loop.

**Figure A3:**
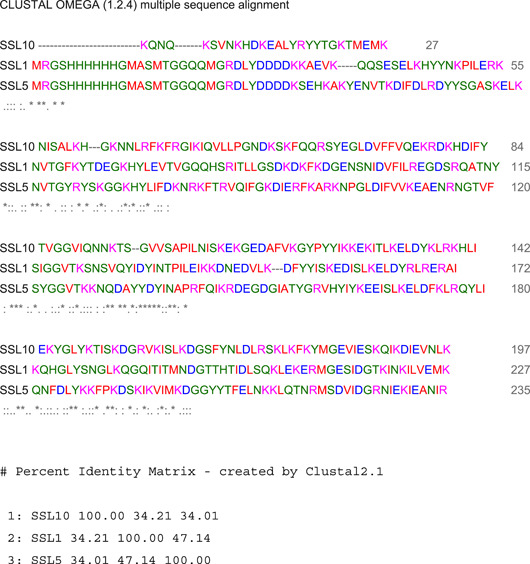
Multiple sequence alignment between the three reviewed SSLs created with Clustal Omega.

**Figure A5:**
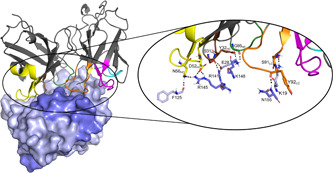
A predicted binding complex of SSL1 with scFv‐93. A crystal structure of SSL1 (PDB ID: 4O1N) was docked to a homology modeled scFv‐93 and the complex was subjected to a 100‐ns MD simulation. The last frame of the simulation is presented here. SSL1 is presented as light blue surface/sticks and the C‐terminal α4β9 domain residues are colored slate blue. The CDR‐loops of scFv‐93 are colored as follows: L1 magenta, L2 cyan, L3 orange, H1 brown, H2 yellow, and H3 green. Interacting residues are labeled and presented as sticks (right). Polar contacts are shown as black dashed lines. MD, molecular dynamics.

**Figure A6:**
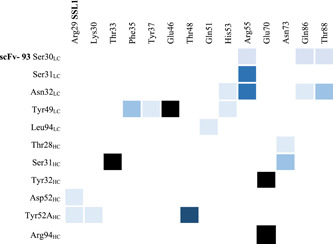
Interacting residues between the scFv‐93 (column) and SSL1 β1‐3 (row) interface. The residues that are in contact during the MD trajectory are shown as squares with different intensities of blue, depending on the frequency of the interaction (light blue for sporadic contacts and the most frequently occurring in black). The data are from the last 50 ns of the simulation collected from every ns of the trajectory. HC, heavy chain; LC, light chain; MD, molecular dynamics.

A cross‐reactive/broad‐range SSL inhibitor would be an ideal antivirulence agent. However, a broad‐range SSL inhibitor might be hard to achieve even though the proteins are homologous and show a similar fold. Since the sequence identity of the SSLs lies at around 20%–60%, with only sporadic residues conserved along the sequence, it would be extremely challenging to find a compound that could bind to and inhibit several of the SSL protein family members simultaneously. In this study, the produced scFv‐clones were tested for cross‐binding toward the three reviewed SSLs. Also, during this study, an attempt was made to obtain antibodies binding and inhibiting all three SSLs, by changing the SSL target for each panning round. However, no antibody was found that could bind to the three targets simultaneously. This is likely due to the low sequence identity between these three SSLs (Figure A3).

Since SSL1 and SSL5 share the same fold, we used the two SSL5 domains that have been experimentally shown to interact with MMP9 (Kohno et al., 2018) as a basis when creating complex models of SSL1 and the inhibitor scFv‐93. We hypothesized that the antibody could block SSL1 from binding to MMP9 by targeting the SSL1 C‐terminal α4β9 domain (β‐grasp) or N‐terminal β1‐3 domain (OB domain). The MD simulations along with the binding free energy calculations supported the scFv‐93 binding site at SSL1 to be located at the N‐terminal β1‐3 domain. We also modeled an alternative complex structure with the antibody binding to the SSL1 C‐terminal α4β9 domain. The binding free energy of this complex was considerably less favorable compared with the N‐terminal binding site. Unlike the SSL5 β1‐3 domain, the SSL5 α4β9 region was not shown to play a role in the inhibition of MMP9 enzymatic activity (Kohno et al., 2018) and thus it may also not be a binding site for the scFv‐93 on SSL1. Further, the monoclonal antibody discovered by Oku et al. (2018) binds to the C‐terminal β‐grasp motif but was found not to interfere with the SSL5 binding to MMP9. In line with this, our modeling and experimental results suggest that it is the SSL1 OB domain, That is, required for the MMP9 inhibitory interaction. However, experimental studies such as deletion/domain swap mutants on the SSL1 or X‐ray crystallographic studies on the whole SSL1‐scFv‐93 complex should be conducted to confirm the exact location for binding.

Finally, both SSL1 and SSL5 are broad‐range MMP inhibitors and thus interfere with bacterial clearance through MMP‐mediated immune functions (Koymans et al., 2016). In addition, SSL1 has the exceptional ability to cause corneal toxicity and function as a protease as a homodimer (46 kDa). Therefore, an antibody able to inhibit SSL1 could contribute towards the development of a novel antivirulence agent to be used in *S*. *aureus‐*caused infections. An SSL1‐inhibiting (antibody) drug could be applied as an adjunct therapy to complement the use of classical antibiotics during *S*. *aureus* infection (e.g., to limit SSL1‐caused tissue damage during an ocular infection) and could allow a more efficient clearance of the bacteria.

## AUTHOR CONTRIBUTIONS

**Ida Alanko**: Conceptualization (equal); formal analysis (lead); funding acquisition (supporting); investigation (lead); validation (equal); writing—original draft (lead); Writing—review and editing (lead). **Rebecca Sandberg**: Formal analysis (equal); investigation (equal); writing—review and editing (equal). **Eeva‐Christine Brockmann**: Methodology (equal); supervision (equal); validation (equal); writing—review and editing (equal). **Carla J. C. Haas**: Investigation (equal); methodology (equal); supervision (equal); validation (equal); writing—review and editing (equal). **Jos A. G. Strijp**: Project administration (equal); resources (equal). **Urpo Lamminmäki**: Conceptualization (equal); methodology (equal); resources (equal); supervision (equal). **Outi M. H. Salo‐Ahen**: Conceptualization (lead); funding acquisition (lead); project administration (equal); resources (equal); supervision (equal); writing—review and editing (equal).

## CONFLICT OF INTEREST STATEMENT

None declared.

## ETHICS STATEMENT

None required.

## Figures and Tables

**Figure A4:**
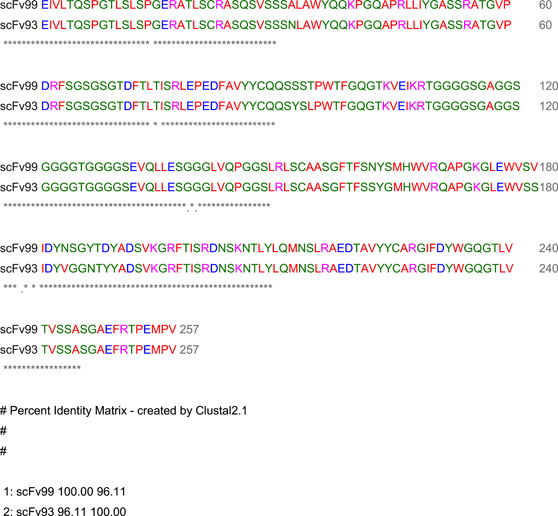
Pairwise sequence alignment of scFv‐93 and scFv‐99 created with Clustal Omega.

## Data Availability

All data generated or analyzed during this study are included in this published article.
